# Polyphenols and Ischemic Stroke: Insight into One of the Best Strategies for Prevention and Treatment

**DOI:** 10.3390/nu13061967

**Published:** 2021-06-08

**Authors:** Francesca Pacifici, Valentina Rovella, Donatella Pastore, Alfonso Bellia, Pasquale Abete, Giulia Donadel, Silvia Santini, Heinz Beck, Camillo Ricordi, Nicola Di Daniele, Davide Lauro, David Della-Morte

**Affiliations:** 1 Department of Systems Medicine, University of Rome “Tor Vergata”, 00133 Rome, Italy; francesca.pacifici@uniroma2.it (F.P.); valerovix@yahoo.it (V.R.); d.pastore3@inwind.it (D.P.); bellia@med.uniroma2.it (A.B.); didaniele@med.uniroma2.it (N.D.D.); d.lauro@med.uniroma2.it (D.L.); 2 Department of Medical Sciences, Fondazione Policlinico Tor Vergata, 00133 Rome, Italy; silvia.santini83@gmail.com; 3 Department of Translational Medical Sciences, University of Naples “Federico II”, 80138 Naples, Italy; p.abete@unina.it; 4 Department of Clinical Science and Translational Medicine, University of Rome “Tor Vergata”, 00133 Rome, Italy; donadel@uniroma2.it; 5 Campus Principe di Napoli, Università Unipegaso, 80132 Napoli, Italy; bmconsulting@heinzbeck.com; 6 Diabetes Research Institute, University of Miami Miller School of Medicine, Miami, FL 33136, USA; ricordi@miami.edu; 7 Department of Neurology and Evelyn F. McKnight Brain Institute, Miller School of Medicine, University of Miami, Miami, FL 33136, USA; 8 Interdisciplinary Center for Advanced Studies on Lab-on-Chip and Organ-on-Chip Applications (ICLOC), University of Rome “Tor Vergata”, 00133 Rome, Italy; 9 Department of Human Sciences and Quality of Life Promotion, San Raffaele Roma Open University, 00166 Rome, Italy

**Keywords:** phytochemicals, nutraceutical, cardiovascular disease, sirtuins

## Abstract

Ischemic stroke (IS) is still among the leading causes of death and disability worldwide. The pathogenic mechanisms beyond its development are several and are complex and this is the main reason why a functional therapy is still missed. The beneficial effects of natural compounds against cardiovascular diseases and IS have been investigated for a long time. In this article, we reviewed the association between the most studied polyphenols and stroke protection in terms of prevention, effect on acute phase, and rehabilitation. We described experimental and epidemiological studies reporting the role of flavonols, phenolic acid, and stilbens on ischemic mechanisms leading to stroke. We analyzed the principal animal models used to evaluate the impact of these micronutrients to cerebral blood flow and to molecular pathways involved in oxidative stress and inflammation modulation, such as sirtuins. We reported the most significant clinical trials demonstrated as the persistent use of polyphenols is clinically relevant in terms of the reduction of vascular risk factors for IS, such as Atrial Fibrillation. Interestingly, different kinds of polyphenols provide brain protection by activating different pathways and mechanisms, like inducing antithrombotic effect, such as Honokiol. For this reason, we discussed an appropriate integrative use of them as a possible therapeutic alternative against stroke.

## 1. Introduction: Polyphenols

Polyphenols are micronutrients present in a variety of foods, which gained interest over the last 30 years due to their antioxidant properties and their emerging role in the prevention of several diseases linked to oxidative stress such as cancer, cardiovascular and neurodegenerative disorders [1]. Polyphenols are also secondary products of plant metabolism, whose main function is to protect organisms from damage by ultraviolet radiation and pathogens [2]. The total dietary daily intake is about 1 g/d, 10 times higher the daily antioxidant intake from Vitamin C and 100 times the one from Vitamin E [3]. The main dietary source of polyphenols are fruits such as apple, grape, cherry, pear, and various berries which contain up to 200–300 mg of polyphenols per 100 g fresh weight, and beverages: a cup of tea or coffee or a glass of red wine contains about 100 mg of polyphenols [3]. Vegetables, cereals, dry legumes, and chocolate also contribute significantly to their daily intake [3].

**Figure 1:**
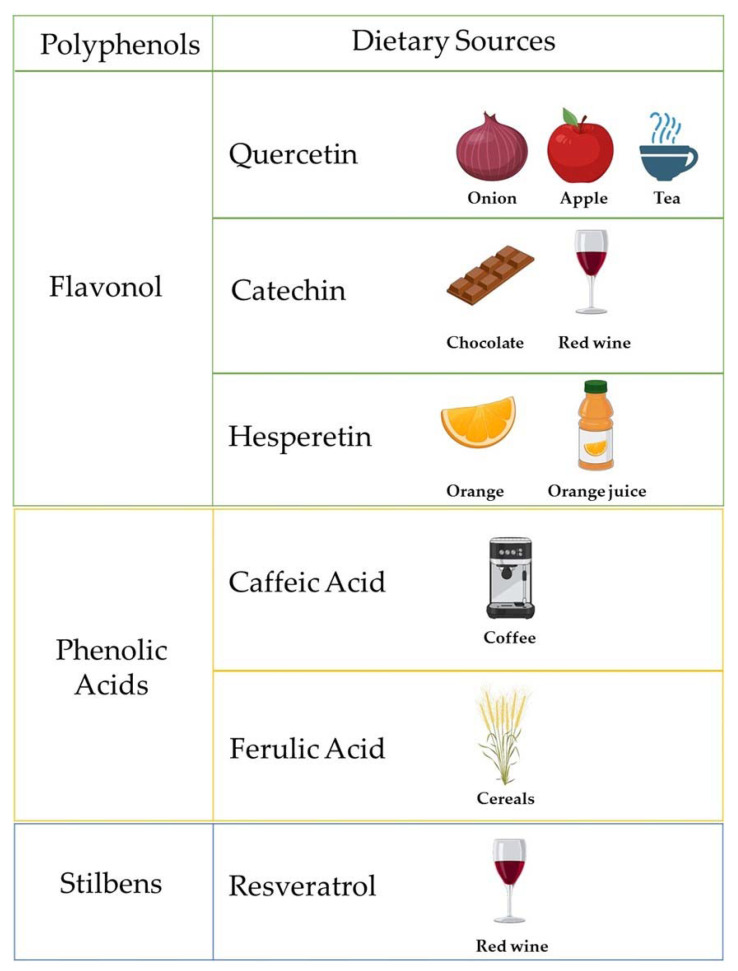
Schematic representation of polyphenols and their related dietary sources. Created with BioRender.com.

Figure 1 reports the most common polyphenols and their dietary sources.

The most relevant scientific evidence reporting the benefic effect of polyphenols on chronic disorders refers to the so-called French paradox [4]. Although French people consume elevated levels of saturated fatty acids, which are generally associated with high mortality due to coronary heart disease, they showed low mortality [4]. The authors suggested that the high red wine consumption (and thus a high amount of resveratrol) resulted in being protective on cardiovascular risk. Accordingly, a low to moderate consumption of alcoholic drinks rich of polyphenols, such as red wine, was associated with a lower risk of cardiovascular events [5], and with a reduced mortality risk in healthy subjects, due to a decrease in coronary events, and also in patients with documented cardiovascular diseases (CVD) [6].

Several clinical and experimental studies demonstrated also that consumption of polyphenol-rich food and beverages increases plasma antioxidant capacity [7,8], and also reduces the DNA oxidative damage [9] and induces an anti-inflammatory and immune-modulating action, explaining, at least in part, their protective role on CVD [10]. In agreement with these findings, an intake of 800 g/d of fruit and vegetables was associated with a linear decrease in CVD risk [11]. Moreover, a Mediterranean dietary pattern composed of food rich in polyphenols, such as olive oil, legumes, fruits, and vegetables, is associated with a lower risk of CVD incidence and mortality [12]. A study conducted by Kokubo Y. and colleagues reported an increased consumption of soy isoflavones in post-menopausal women, which are generally at high risk of developing CVD [13] and was related to a significant reduction in the risk of myocardial and cerebral infarction [14].

The cardioprotective effect of polyphenols, behind their antioxidant properties, may be also linked to their antithrombotic action. Accordingly, extracts from green tea and de-alcoholated red wine inhibited platelet aggregation in in vitro studies [15,16]. Furthermore, it has been demonstrated that several polyphenols blunted endothelial dysfunction, facilitating nitric oxide-mediated vasodilation [17,18,19]. Among polyphenols, resveratrol has been reported to ameliorate endothelial function by decreasing the release of pro-inflammatory cytokines, such as interleukin (IL)-1β and tumor necrosis factor (TNF)α [20,21,22], by reducing oxidative stress in a Sirtuin1-dependent manner [23,24], and by stimulating the biosynthesis of endogenous antioxidant enzymes, such as superoxide dehydrogenase (SOD), catalase and glutathione peroxidase (GPx) [25]. A protection against CVD may be also mediated by the ability of polyphenols to control hormonal synthesis linked with metabolic dysfunction. Recently, we reported that Tyrosol (TR), a major polyphenol found in extra virgin olive oil (EVOO), reduced differentiation in murine 3T3-L1 preadipocytes through downregulation of adipogenic proteins, inflammation, and oxidative stress. Moreover, TR triggered adipose tissue browning throughout the induction of the AMPK-ATGL-UCP1 pathway, suggesting its potential utilization as a treatment against diabetes and CVD prevention [26].

## 2. Ischemic Stroke Physiopathology

### 2.1. Ischemic Stroke Subtypes

Stroke is now the second leading cause of death worldwide [27], and is defined as an impairment in blood supply to the brain [28], and is linked to different cardiovascular dysfunctions [29]. Two main types of stroke exist: ischemic stroke (due to clot-mediated blood vessel occlusion), which accounts for about 85% of all stroke cases, and hemorrhagic stroke (caused by blood vessel rupture), which accounts for 15% of total cases [30]. Following the stroke, while the core region undergoes sudden death, the surrounding injured regions (called penumbra) may be able to recover their functions [28]. In particular, in the core stroke region, the blood perfusion is dramatically impaired (10–12 mL/100g/min), leading to a reduction in the oxygen and glucose supply, with a consequent decrease in energy production and neuronal death [28]. Conversely, blood perfusion in the penumbra is around 60 mL/100g/min, causing neuronal dysfunction. However, whether reperfusion occurs in time, neuronal death is avoided and their function may be recovered [28].

**Figure 2:**
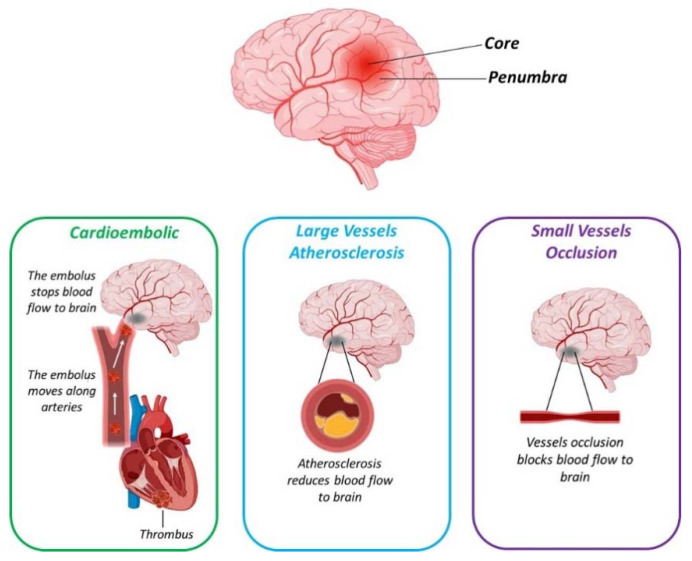
Schematic representation reporting the three main subtypes of ischemic stroke. Created with BioRender.com.

Different subtypes of ischemic stroke exist. Based on the TOAST (Trial of Org 10,172 in Acute Stroke Treatment) [29] classification, in fact, five different subtypes are distinguished (Figure 2): 1. Cardioembolic; 2. Large vessels atherosclerosis; 3. Small vessels occlusion; 4. Unusual cause (atherosclerosis-independent); 5. Undetermined etiology.

Cardioembolic stroke accounts for 25% to 40% of all cerebral infarction worldwide [31]. Compared to the other stroke subtypes, cardioembolic stroke is associated with the worst prognosis for patients [32]. The main risk factor is Atrial Fibrillation (AF), an alteration of the cardiac rhythm that increased with age and is also related to CVD [33].

Atherosclerosis of intracranial or extracranial arteries leads to vessel stenosis, causing a reduction in blood flow to the brain, and consequently to ischemic stroke, accounting for about 20% of all stroke cases [34,35]. The middle cerebral arteries are the main site for intracranial atherosclerosis (ICA) [34]. It has been reported that ICA is directly related to age with an increased risk ranging from 43% in 60 years old age subjects, to 80% in those older than 80 years of age [34].

Small vessel occlusion, or lacunar stroke, accounts for about 25% of stroke cases and involves the deep microcirculation of the brain [36]. Although the infarcted regions are very small compared to other subtypes of stroke, it is very harmful and patients affected by lacunar stroke showed several physical and intellectual alterations [37]. Moreover, it has been reported that about 25% of patients are prone to display a successive stroke episode within 5 years [36].

Strokes with uncommon causes account for a small group of all stroke cases [29]. Among the causes, non-atherosclerotic vasculopathies, artery dissection and prothrombotic disorder have been reported [29,30].

In some stroke cases, defined as cryptogenic or with undetermined etiology, a specific diagnosis is very difficult due to incomplete diagnostic evaluation, confounding causes, or none-specific causes assessed [29]. Thus, a brain infarcted area is recognized but none of the above-mentioned causes can be traced back to this stroke event.

Amongst the undetermined causes of stroke, we can find the embolic stroke of an undetermined source (ESUS), which accounts for an average of 17% of ischemic stroke cases, and where embolism is the leading mechanism [38]. Subjects affected by ESUS are younger compared to those with other ischemic stroke subtypes [38]. Moreover, there is an average of 4.5% annual recurrence of stroke in these patients, suggesting the relevance of a more appropriate antithrombotic prophylaxis.

### 2.2. Role of Oxidative Stress in the Pathophysiology of Ischemic Stroke

Increased levels of reactive oxygen species (ROS), which contribute to enhance oxidative stress, have been reported during ischemic stroke [39]. In particular, compared to other organs, the brain resulted in being highly vulnerable to oxidative stress, since the lower presence of intracellular antioxidants levels and the presence of a large amount of intracellular lipids that can be oxidized by ROS [39]. Reduced levels of endogenous antioxidants were also reported in humans following ischemic stroke [40]. After their release, ROS interact with several biological molecules such as protein, lipids and DNA, leading to an alteration of their structure and functions [39]. In particular, lipid peroxidation, protein denaturation, and DNA modifications are among the most common consequences of ROS accumulation [39].

Lipid peroxidation is the more harmful compared to protein oxidation, since following this initial step, a self-propagation of the oxidative process is activated, leading to an enhancement of oxidative damage [41]. An increase in proteins and lipid oxidation has been reported in both stroke patients and in mice models of stroke, such as middle cerebral artery occlusion (MCAO) [41,42,43,44]. The most relevant markers of lipid peroxidation are malondialdehyde (MDA) and 4-hydroxynonenal (4-HNE) [45,46]. Accordingly, a significant increase in MDA levels were found both at 24 h and 7 days post-acute ischemic stroke patients, in association with reduced levels of antioxidant enzymes [47]. Moreover, stroke-prone hypertensive rats, which developed lethal stroke, showed a dramatic increase in 4-HNE levels compared to the control group [48].

During stroke, a perturbation in the endoplasmic reticulum (ER) function occurs, leading to an unfolded protein response (UPR) with impaired protein function [49]. A class of protein responsible for reducing UPR was composed of the heat shock proteins (HSP) [50]. Among these, in particular, HSP70 has a pivotal role in neuronal protection following ischemic insult [50]. In an experimental model of focal ischemia, in fact, it was suggested that was an increase in HSP70 levels in the penumbra, where the neurons more resistant to ischemia are localized [51]. Accordingly, in mice, following cerebral focal ischemia, HSP70 injection of reperfusion displayed a reduction in infarct lesion and better neuronal outcomes simultaneously [52].

DNA modification is another important consequence of increased ROS during IS [39]. ROS, in fact, enhances DNA methylation levels in several animal models of ischemia and also in stroke patients [53]. This phenomenon is associated with an increase in ischemic injury, endothelial damage stroke occurrence, and cerebral infarction, among others [53]. Accordingly, the administration of methylation inhibitors is able to reduce brain damage in ischemic animal models [54,55]. Another modification observed in the animal model of IS is a decrease in several histones’ acetylation, leading to more severe neuronal damage and enhanced oxidative stress [53,56]. Histone deacetylase (HDACs) inhibitors improved the antioxidant response following ischemia [57]. Moreover, HDAC1 and 2 were more active in the penumbra, according to the presence of less vulnerable neurons to stroke, while their levels were decreased in the core of lesions, where more oxidative stress and neuronal death occur [56].

### 2.3. Role of Inflammatory Cells in the Pathophysiology of Ischemic Stroke

Following IS, morphological and functional alteration of cells, such as microglia, in the infarcted area occurs, leading to the activation of a pro-inflammatory cascade [58]. In particular, microglia assumed an ameboid phenotype able to attract inflammatory cells [59]. Microglia activation leads to both pro- [58] and anti-inflammatory effects [60]. Once microglia is activated, an increase in ROS, pro-inflammatory cytokines and metalloprotease was observed, which in turn contribute to the severity of brain injury [58,61]. According to these findings, inhibition of microglia activation by using 2% isoflurane preconditioning markedly reduced inflammation and cells apoptosis in the penumbra, contributing to a decrease in MCAO-induced infarct lesion [62]. However, an impairment in microglia activation has been associated to increased infarct size and neuronal death following MCAO in mice [63]. This can be explained, at least in part, by the fact that microglia release several neurotrophic factors, such as Transforming Growth Factor Beta (TGFβ), and several anti-inflammatory cytokines, like IL-6 [64]. Moreover, microglia’s neuroprotective effect is also associated with a phenomenon referred to as capping: following neuronal injury, microglia closely associates with damaged neurons leading to their phagocytosis and contributing to the reduction in infarct size [58].

Similarly to microglia, astrocytes also showed a double role following ischemia [65]. Astrocyte activation, after neuronal injury, enhance the release of several pro-inflammatory cytokines, such as TNFα and IL-1β, leading to an increase in oxidative stress, in neuronal death and to a reduction in neurogenesis [66,67,68]. At the same time, astrocytes produced neurotrophic factors, such as brain-derived neurotrophic factor (BDNF), which confers neuroprotection [58]. Accordingly, the conditioned media of astrocytes following MCAO reduced the infarcted lesion, suggesting astrocytes-mediated delivery of neuroprotective factors during brain injury [69]. Moreover, astrocytes structurally maintain the blood–brain barrier (BBB) architecture. However, the metalloprotease released during ischemia by other cells like microglia, disrupted the connection between astrocytes and BBB, leading to its alteration and promoting the infiltration of inflammatory cells in the injured area [58,70].

Other important inflammatory cells that have harmful effects following ischemia, are leukocytes [58]. In particular, neutrophils are the first cells invading the damaged brain area, enhancing the release of pro-inflammatory cytokines, ROS production and BBB permeability, boosting the ischemic injury [71]. Moreover, leukocyte activation induced also platelet aggregation and microvasculature obstruction, leading to a reduction in blood flow to the injured brain region, dramatically potentiating the ischemic damage [58].

## 3. How Polyphenols Target Preventive Pathways against Ischemic Stroke

### 3.1. Epidemiological Studies

As previously reported, AF increased the risk of ischemic stroke from 4 to 5 folds [72]. A healthy dietary regimen has been proposed to reduce AF and its related CVD, such as stroke [73]. In particular, a Mediterranean diet, rich in polyphenols, significantly impacts on the incidence of IS [74,75]. In agreement, a PREDIMED (Prevención con Dieta Mediterránea) clinical trial reported the Mediterranean diet to reduce the incidence of stroke and myocardial infarction [73]. A Mediterranean diet enriched in extravergin olive oil (EVOO, which contains polyphenols) significantly reduced the risk of AF [73]. The authors hypothesized that this protection against AF may be attributable to the well established anti-inflammatory and anti-oxidant properties of polyphenols present in EVOO [73].

A longitudinal study conducted in a large population (30,239 subjects) belonging to the REasons for Geographic And Racial Differences in Stroke (REGARDS) study, aimed to analyze the association between thy Mediterranean diet and incidence of IS [74]. The obtained results showed that a high adherence to the Mediterranean diet was associated with a lower risk of IS but not haemorrhagic stroke [74]. The authors suggested that this difference may be associated with the protective effect of the Mediterranean diet against common risk factors for IS such as diabetes mellitus and metabolic syndrome compared to those more associated with a risk of hemorrhage [74]. Moreover, it was also suggested that a Mediterranean diet is associated with a lower progression of atherosclerosis, in particular with carotid intima media thickness (IMT) [76], which is considered to be a well-established risk for diabetes mellitus and for IS rather than haemorrhagic stroke [76,77]. In a prospective study conducted on 74,961 Swedish women and men, the effects of black tea on stroke risk was evaluated [78]. The collected results highlighted that 4 or more cups of black tea daily were inversely correlated with stroke risk [78].

Taken together, all this evidence suggests that polyphenol consumption may be linked to a reduction in stroke events. Nevertheless, further studies are needed to deeply understand the molecular mechanisms underlying polyphenol-mediated protection against stroke.

Data reported are summarized in Table 1.

### 3.2. Animal Models Reporting the Positive Effects of Polyphenols against Ischemic Stroke

The neuroprotective effects of polyphenols on IS have been also evaluated by several animal models. Resveratrol is the most common polyphenol present in grapes and red wine [83], and is most studied against CVD. The effect of this compound has been widely investigated in different models. It has been reported as resveratrol administration in rats, and exerts a protective effect against stroke by activating the neuroprotective pathway mediated by sonic hedgehog (Shh) [83,84]. In particular, 7 days of resveratrol pretreatment before MCAO significantly reduces infarct size and improves neurological function in rats [83]. Moreover, resveratrol also decreased neuronal death by activating the neuroprotective pathway mediated by Shh both in rats following MCAO and in cortical neuron culture subjected to oxygen-glucose deprivation (OGD) [83]. We previously reported on resveratrol-injected i.p. by activating the SIRT1-UCP2 pathway, which significantly increased the neuronal mitochondria respiration reflecting an enhanced ATP synthesis efficiency and in turn inducing brain protection against ischemia [85].

A study analyzed the role of green tea extract (GTx) and epigallocatechin gallate (EGCG) in rats following the MCAO procedure [79]. In particular, GTx and EGCG were administered either before MCAO for 7 consecutive days. The results demonstrated that both these polyphenols reduced the spatial and reference memory loss induced by ischemic damage [79]. Moreover, a significant decrease in brain infarct size was observed in treated animals [79]. At the molecular level, authors found that GTx and EGCG blunted lipid peroxidation, which was enhanced following MCAO [85]. In association, increased levels of the antioxidant enzyme glutathione was reported, suggesting that polyphenols may exert a neuroprotective effect against IS through antioxidant properties.

In addition to reduced infarct size and increased glutathione levels, improved neurological scores and reduced apoptotic neuronal death have been reported for EGCG [80]. Furthermore, the authors showed a significant enhancement in nuclear factor erythroid-2 related factor 2 (Nrf2) expression following EGCG administration [80]. Since Nrf2 antioxidant activity has been previously reported to play a pivotal role against stroke injury [86], the authors suggested that EGCG neuroprotective effects may be mediated by induction of Nrf2.

Pomegranate polyphenols have been demonstrated to positively impact on several pathological conditions, such as diabetes, atherosclerosis, hyperlipidemia and cancer [87,88,89,90]. The effect of pomegranate against stroke was also evaluated [81]. In particular, pomegranate extracts were administered for two weeks before inducing MCAO in rats [81]. Then, active and passive avoidance memory deficits were assessed, showing as pomegranate and significantly improving both of them. This study also suggested pomegranate-ameliorated behavioral deficits due to IS by crossing the BBB. However, the molecular mechanisms driving this benefic effect is still unclear.

Another study reported the neuroprotective effect of salvianolic acid B against stroke [82]. Salvianolic acid B is one of the polyphenolic compounds present in Salvia miltiorrhiza [82]. The study showed as pre-treatment with this polyphenol in rats subjected to ischemic insult significantly decreased infarct volume and improved neurological scores [82]. Moreover, an anti-inflammatory effects was also reported in association with increased expression levels of Silent information regulator 1 (SIRT1) [82]. These findings suggested that salvianolic acid B may reduce brain injury by activating the neuroprotective pathway mediated by SIRT1 as well as resveratrol.

All data reported are summarized in Table 1.

## 4. The Polyphenols Therapeutic Utilization against Acute Ischemic Stroke and on Stroke Rehabilitation

### 4.1. Epidemiological Studies

Following the stroke event, survivors may show cognitive impairment and functional disabilities [91]. Now, there is not an approach able to fully recover neuronal damage after stroke, impacting dramatically on individual independence, but also on public health costs. Therefore, it is mandatory to find novel theraputic targets and approaches to prevent and/or cure stroke, in order to improve neuroprotection, reducing the harmful effect induced by this disabling disease.

However, as will be deeply discussed in the following paragraph, it has been reported that in animal models subjected to ischemia, a polyphenols-enriched diet may promote neuronal recovery. Particularly, pomegranate polyphenols displayed a protective role after ischemia, and chronic disorders such as hypertension, diabetes and CVD in animal models [88,92]. In a blinded-randomized clinical trial, pomegranate polyphenols or placebo pills were administered 2 weeks after a stroke event for 1 week [92]. The rehabilitative period for the two groups was different, with a lower time for the treated group compared to the placebo group. Moreover, pomegranate administration improved cognitive and functional parameters [92]. Interestingly, locomotion reached the highest score. Taken together, these results suggested that pomegranate may be useful for post-stroke rehabilitation in order to improve cognitive and functional recovery. However, few subjects were enrolled in this study and further trials involving the highest number of patients is mandatory to confirm these relevant results.

Following acute IS, the most common pharmacological treatment adopted is the recombinant tissue plasminogen activator (rt-PA), which degraded the fibrin coat, restoring the blood flow in the ischemic regions [93]. However, the treatment must be administered within 3 h from an ischemic event, otherwise fewer effects are reported in association with side effects such as intracerebral hemorrhage [94]. Interestingly, it has been demonstrated that the tandem administration of rtPA and polyphenols may increase this time window by enhancing the possibility for intervention against stroke. In a clinical study, both resveratrol or a placebo were administered in combination with rtPA in subjects with regular or delayed post-stroke treatment [95]. Twenty-four hours following administration, stroke outcomes were assessed by using the NIH stroke scale (NIHSS). Subjects belonging to the resveratrol + rtPA group showed a significant improvement in NIHSS scores in association with reduced levels of metalloproteinase (MMP)-2 and 9 (which contributes to neuronal damage following hypoxia) [95,96]. Similar results were also obtained by using EGCG [97] or fisetin [98] in association with rtPA. All these findings suggest that polyphenols may be useful to enlarge the therapeutic window for acute stroke patients leading to an improvement of stroke-related outcomes.

All data reported are summarized in Table 2.

### 4.2. Animal Studies

Here we have already reported the benefic effect of salvianolic acid pre-treatment against stroke [82]. In a subsequent study, it was evaluated whether salvianolic acid for injection (SAFI) could exert protection also following distal MCAO (dMCAO) [99]. In particular, mice were subjected to dMCAO and SAFI was administered for 14 days starting 24 h post dMCAO. Results showed an improvement in behavioural tests, partially due to the SAFI-mediated activation of the Shh pathway, leading to neurogenesis and brain injury recovery. In agreement with the benefic effect of salvianolic acid, another study, showed in mice subjected to dMCAO, post-stroke treatment with this phenolic compound induced angiogenesis in a JAK2/STAT3 (Janus kinase 2/signal transducer and activator of transcription 3)-dependent manner, leading to improved functional recovery [102]. All these findings suggested that salvianolic acid may be considered as a therapeutic strategy for stroke recovery.

Following IS, an increase in inflammation occurs, contributing to brain damage [103]. Targeting inflammation following stroke may then be considered a strategy to confer neuroprotection. Accordingly, resveratrol administration following MCAO in rats blunted neurological deficits and cerebral edema [100]. Moreover, a significant decrease in inflammation and inflammatory mediators was also observed [100]. Interestingly, the administration of a phosphatidylinositol 3-kinase/Akt (PI3K/Akt) inhibitor abolished all these benefic effects [100], suggesting that the neuroprotection exerted by resveratrol may be, at least in part, mediated by the activation of the anti-apoptotic and antioxidant PI3K/Akt pathway.

In a recent study, it has been demonstrated that resveratrol administration following a stroke event may exert a neuroprotective effect against stroke by regulating the gut–brain axis [101]. In particular, the study reported as resveratrol induced the polarization of T lymphocyte from the Th1 pro-inflammatory to the Th2 anti-inflammatory phenotype, blunting small-intestine inflammation and reducing vascular permeability. All these regulations in the inflammatory condition ultimately led to a decrease in cytokine-mediated BBB and related brain damage. Therefore, it has been speculated that the gut–brain axis is a novel therapeutic target for ischemic stroke and shed a new light for a novel relevant action of resveratrol against stroke.

All data reported are summarized in Table 2.

## 5. Combination of Different Polyphenols as a Novel Therapeutic Strategy against Ischemic Stroke

Based on the findings previously reported in this article, it was possible to highlight the power of polyphenols against IS in the acute phase rather than in the phase of rehabilitation after stroke. This particular power is linked to the pleiotropic effects that many polyphenols present against IS, especially, as we have mentioned, through anti-inflammatory and antioxidant effects. Interestingly, different polyphenols act in different molecular pathways to regulate these processes and, therefore, the multiple use of them, in a systematic approach, may be useful in boosting their effect against vascular injury. Several researchers, as well as several pharmaceutical companies, are trying to find the best composition of polyphenols to maximize their effect and to take advantage from the combination.

For instance, resveratrol has been shown to mimic the effect of ischemic preconditioning, the most powerful endogenous mechanism of protection against ischemia, via SIRT1/UCP2 activation [85]. Therefore, the right doses of resveratrol may be used for preventive strategies. Moreover, the problem of its bioavailability may be bypassed by the use of other types of polyphenols, like pterostilbene [104], which has been demonstrated to also have an effect against stroke [105] and in sirtuins activation [106]. The same may be accounted for polydatin, which is another polyphenol able to either activate sirtuins [107] or than protect it against IS [108].

Moreover, in the acute phase, as showed in Figure 2, the major problem during IS is the clot formation that may be prevented and counteracted by antiaggregant and anticoagulant strategies. Polyphenols, such as honokiol, a phenolic compound isolated from the root and bark of *Magnolia officinalis,* has been proven to be one of the most powerful natural antiaggregants through the inhibition of 5-HT secretion [109]. Moreover, through this antithrombotic effect, honokiol showed protection against stroke in animal models [110] and has been already suggested as a potential therapeutic strategy in humans [111].

Therefore, based on this evidence, a unique compound including all these polyphenols that act at different levels and in different molecular pathways in terms of prevention and treatment against IS may be helpful. Some laboratories are exploring these studies in other fields of research, like we are doing with worldwide collaborations with a novel compound called A6 in diabetes, dementia, aging, and infective diseases [112]. However, therapy for stroke would certainly benefit this approach in the near future.

## 6. Conclusions

Stroke is still among the leading causes of mortality and disability worldwide. The saddest thing is that even with the huge amount of research and funding spent on trying to find a cure, so far, we are still fighting against time, procedures, and concomitants. In the meantime, while science aims to come up with the definitive therapy, the real answer may be in nature, which can help us in achieve greater prevention. This may be based on controlling vascular risk factors, through a Mediterranean diet enriched in EVOO and resveratrol, and though supplementation with compounds targeting specific antioxidant and anti-inflammatory pathways, which are pivotal in ischemic brain damage after stroke.

## Figures and Tables

**Table 1:** Epidemiological Studies showing beneficial effects of polyphenols against Ischemic Stroke.

Treatment	Human	Animal	Effect	References
**Mediterranean Diet and EVOO (PREDIMED study)**	X		↓ Incidence of stroke ↓ Myocardial infarction ↓ AF	[73]
**Mediterranean Diet (REGARDS study)**	X		↓ Ischemic stroke ↓ Progression of carotid intima media thickness ↓ Diabetes	[74,76,77]
**Black Tea (4 or more cups)**	X		↓ Stroke risk	[78]
**Resveratrol**		X	↓ Infarct size ↓ Neuronal death ↑ Neurological function ↑ Shh pathway	[79,80]
**Green tea extract and EGCG**		X	↓ Spatial and reference memory loss ↓ Lipid peroxidation ↓ Infarct size	[79]
**EGCG**		X	↓ Infarct size ↑ Glutathione levels ↑ Neurological function ↓ Neuronal death ↑ Nrf2 levels	[80]
**Pomegranate**		X	↑ Memories deficits due to MCAO	[81]
**Salvianolic acid B**		X	↓ Infarct size ↑ Neurological function ↓ Inflammation ↑ SIRT1 expression	[82]

↑ = increase and improve; ↓ = reduce.

**Table 2:** Polyphenols-mediated beneficial outcomes and pathways following Ischemic Stroke.

Treatment	Human	Animal	Effect	Reference
**Pomegranate polyphenols**	X		↑ Cognitive and functional parameters	[92]
**Resveratrol, or EGCG, or Fisetin**	X		↑ Therapeutic window ↑ NIHSS scores ↓ MMP-2 and MM-9 levels	[95,96,97,98]
**Salvianolic acid**		X	↑ Behavioural tests ↑ Shh pathway leading to neurogenesis ↑ Angiogenesis mediated by JAK2/STAT3 pathway	[99]
**Resveratrol**		X	↓ Neurological deficits ↓ Cerebral edema ↓ Inflammation ↑ Th2 anti-inflammatory response	[100,101]

↑ = increase and improve; ↓ = reduce.
